# 4D label-free quantitative proteomics analysis to screen potential drug targets of Jiangu Granules treatment for postmenopausal osteoporotic rats

**DOI:** 10.3389/fphar.2022.1052922

**Published:** 2022-11-01

**Authors:** Haiming Lin, Wei Zhang, Yashi Xu, Zexing You, Minlin Zheng, Zhentao Liu, Chaoxiong Li

**Affiliations:** ^1^ College of Integrated Chinese and Western Medicine, Fujian University of Traditional Chinese Medicine, Fuzhou, China; ^2^ College of Pharmacy, Fujian University of Traditional Chinese Medicine, Fuzhou, China; ^3^ Department of Orthopedics, Fuzhou Second Hospital Affiliated to Xiamen University, Fuzhou, China; ^4^ The Third Clinical Medical College, Fujian Medical University, Fuzhou, China; ^5^ Fujian Provincial Clinical Medical Research Center for First Aid and Rehabilitation in Orthopaedic Trauma (2020Y2014), Fuzhou, China

**Keywords:** 4D label-free quantitative proteomics, postmenopausal osteoporotic, OVX rats, Jiangu granules, osteoblast differentiation

## Abstract

**Background:** Postmenopausal osteoporosis (PMOP) is a disease with a high prevalence in postmenopausal women and is characterized by an imbalance in bone metabolism, reduced bone mass, and increased risk of fracture due to estrogen deficiency. Jiangu granules (JG) is a compound prescription used in traditional Chinese medicine to treat PMOP. However, its definitive mechanism in PMOP is unclear. This study used a 4D label-free quantitative proteomics method to explore the potential therapeutic mechanism of JG in an ovariectomy (OVX) rats’ model.

**Materials and methods:** A rat model of PMOP was established by removing the ovaries bilaterally. Nine 3-month-old specific-pathogen-free female SD rats. The nine rats were randomly divided into 3 groups (*n* = 3 in each group): the sham-operated group (J), the ovariectomy group (NC), and the JG treatment (ZY) group. Proteins extracted from the bone tissue of the lumbar spine (L3, L4) of three groups of rats were analyzed by 4D label-free quantitative proteomics, and proteins differentially expressed after JG treatment and proteins differentially expressed after de-ovulation were intersected to identify proteins associated with the mechanism of PMOP by JG treatment.

**Result:** There were 104 up-regulated and 153 down-regulated differentially expressed proteins (DEPs) in the J group vs. NC group, 107 up-regulated and 113 down-regulated DEPs in the J group vs. ZY group, and 15 up-regulated and 32 down-regulated DEPs in the NC group vs. ZY group. Six potential target proteins for JG regulation of osteoblast differentiation in OVX rats were identified by taking intersections of differential proteins in the J group vs. NC group and NC group vs. ZY group.

**Conclusion:** JG may exert therapeutic effects by modulating the expression levels of target proteins associated with osteoblast differentiation to enhance osteoblast differentiation in OVX rats. These results further uncovered the target proteins and specific mechanisms of JG in treating PMOP, providing an experimental basis for the clinical application of JG in treating PMOP.

## Introduction

Postmenopausal osteoporosis (PMOP) is a global public health problem with a high prevalence. It is frequently seen in postmenopausal women due to estrogen deficiency and increased calcium and bone loss due to enhanced osteoclastogenesis and bone resorption with advancing age (Thaung Zaw et al., 2018; Consensus, 1993.). The disease tends to occur in middle-aged and elderly women over 50 years old, with a prevalence of about 50%. About 70% of them will have pathological fractures, and the most common fracture sites are the spine, wrist, and hip. The hip fracture may cause loss of mobility and independent living ability of patients and even death (Anastasilakis et al., 2020; Anupama et al., 2020).

As PMOP progresses, there is a dampening of osteogenic activity, resulting in the loss of bone mass due to the disruption of the osteoblastic balance. However, as the disease progresses, osteogenic activity is suppressed, leading to a disruption of the balance of osteogenesis and osteolysis, resulting in a loss of bone mass (Gimble and Nuttall, 2012; Bidwell et al., 2013; Forostyak et al., 2013).

A traditional Chinese medicine, Jiangu granules (JG) have shown great efficacy in the clinical treatment of PMOP (Huang et al., 2017; Sun et al., 2022). However, the molecular mechanism behind the therapeutic effect remains unclear. The chloroform-extracted part of JG can significantly increase the expression of osteoblast differentiation markers and proteins *in vitro* and promote osteoblast differentiation; it can also significantly promote the proliferation of osteoblasts *in vitro* (Zhou et al., 2022). Although it has been shown that JG can promote osteoblast differentiation by reducing the expression of miR-14 and CylinD1 (Wu et al., 2010; Zhang et al., 2018; Yang et al., 2019; Zhang et al., 2020), its mechanisms of action are still not fully explored, and more in-depth specific mechanisms are needed to investigate the role played by JG in therapy in the clinical setting. Therefore, research to find the mechanisms involved in the regulation of osteoblast differentiation by JG is urgently needed.

4D label-free technology has been applied to proteomics analysis due to its high sensitivity and bioinformatics advantages (Meier et al., 2018). This study aimed to obtain the proteomic profile of OVX rats treated with JG through a 4D label-free based approach and bioinformatics analysis. We conducted a 4D label-free quantitative proteomics study on vertebral tissue samples from three groups of rats to investigate the mechanism of action of JG on osteoblasts in ovariectomized (OVX) rats and to provide a new theoretical basis for the prevention and treatment of PMOP with JG.

In our study, six target proteins in PMOP after JG treatment were identified by analyzing differential proteins from three groups of rats and taking the intersection of proteins from the J group vs. NC group and the NC group vs. ZY group. By bioinformatic analysis of the differential proteins, we found that three of the six differential proteins may affect bone metabolism by influencing osteoblast differentiation, namely l-lactate dehydrogenase B chain (Ldhb), Serine protease inhibitor A3N (Serpina3n) and Leukocyte-specific transcript 1 (Lst1); while the specific mechanisms of Serglycin (Srgn), Fibrinogen beta chain (Fgb) and Hermansky-Pudlak syndrome 5 protein homolog (Saa4) in the regulation of osteoblast differentiation still need to be further investigated.

In summary, we identified six target proteins that are regulated in JG treatment of PMOP, and three of the key proteins may be critical for JG regulation of osteoblast differentiation. The above results provide new evidence to explain the regulation of osteoblast differentiation by JG and provide new ideas for future research on specific mechanisms.

## Material and methods

### Drugs and reagents

JG consists of ten traditional Chinese medicines, including Yin yanghuo as *Epim* *edium brevicornu Maxim. [Berberidaceae; Epimedii Folium]*, Gu suibu as *Davallia mariesii Moore ex Bak. [Davalliaceae; Drynariae Rhizoma]*, Shan zhuyu as *Cornus officinalis Sieb. et Zucc. [Cornice; Corni Fructus]*, Dang shen as *Codonopsis pilosula (Franch.) Nannf. [Campanulace; Codonopsis Radix]*, Shan yao as *Dioscorea opposita Thunb. [Dioscoreaceae; Dioscoreae Rhizoma]*, Chen pi as *Citri [Rutaceae; Citri Reticulatae Pericarpium]*, Xi honghua as *Crocus sativus L. [Iridaceae;Croci Stigma]*, Jiang huang as *Curcuma longa L. [zingiberaceae; Curcumae Longae Rhizoma]*, Gou qi as *Lycium chinense Mill. [Solanaceae; Lycii Fructus]*, and Lu xiancao as *Pyrola calliantha H. Andr. [Pyrolaceae; Pyrolae Herba]*. The original botanical drugs were purchased from Fujian Province Pharmaceutical Company (Fuzhou, China) (Huang et al., 2017; Sun et al., 2022). All components were certified by botanical drugs identification professor with the standard shown in Supplementary Material S1. The voucher specimens were kept in the Qiuzhen Building of Fujian University of Traditional Chinese Medicine at constant temperature and humidity. The details of the specific ingredients and contents of the JG dosage are shown in Table 1. All ingredients were processed and prepared into Chinese botanical granules at one time by the Fujian Institute of Traditional Chinese Medicine (Fujian, China). All the ingredients were soaked in 500 ml of water for 30 min, and 500 ml of water was added. The mixture was decocted at 300°C for 50 min and decocted at 100°C for 30 min then filtered. The extract was concentrated in a vacuum water bath at 60°C heating with a rotary evaporator to 80 ml and stored at 4°C.

**TABLE 1:** Constituents of Jiangu granule formulation.

Chinese name	Latin name	Medicinal parts	Production methods	Season of harvesting	Batch number	Voucher specimen number	Percentage (%)
Yinyanghuo	*Epimedil Folium*	Leaves	Dried	Spring and Summer	20200701	FJTCM-IMA-06001	13.2
Gusuibu	*Drynariae Rhizoma*	Root and rhizome	Dried	Throughout the year	200601	FJTCM-IMA-06002	13.2
Shanzhuyu	*Corni Fructus*	Pulp	Dried	Junction of winter and autumn	191101	FJTCM-IMA-06003	9.9
Dangshen	*Codonopsis Radix*	Root	Dried	autumn	106202003	FJTCM-IMA-06004	11
Shanyao	*Dioscoreae Rhizoma*	root	Dried	Winter	200201	FJTCM-IMA-06005	9.9
Gouqizi	*Lycii Fructus*	Fruit	Dried	Summer and Autumn	200201	FJTCM-IMA-06006	9.9
Chenpi	*Citri Reticulatae Pericarpium*	Pericarp	Dried	Junction of winter and autumn	200501	FJTCM-IMA-06007	6.6
Luxiancao	*Pyrolae Herba*	Whole herb	Dried	Throughout the year	200626	FJTCM-IMA-06008	13.2
Xihonghua	*Croci Stigma*	Flower	Dried	Autumn	20063001	FJTCM-IMA-06009	2.2
Jianghuang	*Curcumae Longae Rhizoma*	Root and rhizome	Dried	Winter	200726	FJTCM-IMA-06010	11

In a previous study by our group, the analysis of the main components of JG using High Performance Liquid Chromatography revealed the following five components in the extract of JG: morroniside (8.77 min) from Shan zhuyu, loganin (12.21 min) from Shan zhuyu, naringin (23.13 min) from Chen pi, hesperidin (23.57 min) from Chen pi, and icariin (29.65 min) from Yin yanghuo (Sun et al., 2022). The source organisms of these five components were identified by the Traditional Chinese Medicine Systems Pharmacology Database and Analysis Platform (TCMSP) which is a unique systems pharmacology platform of Chinese botanical drugs that includes chemicals, targets and drug-target networks, and associated drug-target-disease networks etc, (Ru et al., 2014) (http://sm.nwsuaf.edu.cn/lsp/tcmsp.php). The chemical composition of the drug in JG complies with the ConPhyMP statement and has been validated for classification at “http://www.plantsoftheworldonline.org” (Rivera et al., 2014; Heinrich et al., 2020; Heinrich et al., 2022).

### Animal culture

Nine 3-month-old female SD rats without specific pathogens were randomly divided into three groups (*n* = 3 per group): the sham-operated group (J), the ovariectomy group (NC), and the JG treatment (ZY) group. The PMOP mouse model was established by ovariectomy: 2% pentobarbital sodium was injected intraperitoneally at 30 mg/kg, a median abdominal incision was made, the left and right ovaries were explored bilaterally along the uterus, and the abdominal wall was sutured layer by layer after ovariectomy. Except for the J group, all groups were modeled as above and underwent bilateral oophorectomy, while the J group did not have ovaries removed. The gavage dose of JG was converted according to the body surface area of humans and rats, and the normal saline and drug dosages were the same. The specific formulae are included in Supplementary Material S2. It should be clear that although our dose is calculated according to the normal dose of JG and has been proved to be effective, the potential high dose interference should be considered. The rats were weighed every week, and the gavage dosage was adjusted. In the J group, 7.044 ml/kg/d of saline was administered as gavage; in the NC group, 7.044 ml/kg/d of saline was administered as gavage; in the ZY group, 7.044 ml/kg/d of JG solution was administered as gavage. The rats were weighed every week, and the gavage dosage was adjusted. During the treatment, the rats had free access to food and water. After 12 weeks of the treatment, three rats in each group were injected with an overdose of pentobarbital (40 mg/kg). Vertebrae and serum were collected from all animals. The animal study was reviewed and approved by Department College of Integrated Chinese and Western Medicine, FJTCM.

### Protein extraction

Proteomic analysis was performed on three rat lumbar vertebrae (L3, L4) tissue samples from each group. Bone tissue samples were ground into cell powder using liquid nitrogen and transferred to centrifuge tubes, to which four volumes of lysis buffer containing a mixture of 8 M urea and 1% protease inhibitor were added, followed by three treatments with a high-intensity ultrasonic processor (Scientz). This was followed by centrifugation at 12,000 g for 10 min at 4°C. After removing the remaining debris, the supernatant was collected and the protein concentration of the three sets of samples was determined using the Bicinchoninic Acid Assay (BCA) kit.

### Trypsin digestion

The protein solution from the extracted rat vertebral tissue samples was subjected to the following procedure: dithiothreitol (DTT) at a concentration of 5 mM was added to the protein solution at 56°C. After the reduction reaction had been carried out for 30 min, iodoacetamide (IAA) at a concentration of 11 mM was added in the dark and the alkylation was continued for 15 min at room temperature. At the end of the above reaction, the protein sample was diluted with 100 mM trimonium bicarbonate (TEAB) to make the urea concentration in the protein solution less than 2 M. Finally, trypsin was added to the solution at a ratio of 1:50 (trypsin to protein mass ratio) for first enzymatic digestion overnight, followed by a second 4-h enzymatic digestion at a ratio of 1:100 (trypsin to protein mass ratio).

### Liquid chromatography-tandem mass spectrometry analysis-4D Mass Spectrometer.

The peptides were separated using a NanoElute Ultra Performance Liquid Chromatography system after solubilization using liquid chromatography mobile phase A (aqueous solution containing 0.1% formic acid and 2% acetonitrile). The gradient settings were: 0–70 min, 6%–24% B; 70–84 min, 24%–35% B; 84–87 min, 35%–80% B; 87–90 min, 80% B. The flow rate was maintained at 450 nL/min, where mobile phase B was a solution containing 0.1% formic acid and 100% acetonitrile. The separated peptides from the UHPLC system were injected into the Capillary ion source for ionization and then into the timsTOF Pro mass spectrometer for analysis. The ion source voltage was set at 1.65 kV and the peptide parent ions and their secondary fragments were detected and analyzed using a high-resolution TOF. The secondary mass spectrometry scan range was set to 100–1700 and the data acquisition mode was set to Parallel Accumulated Serial Fragmentation (PASEF) mode. A primary mass spectrum acquisition was followed by 10 PASEF mode acquisitions of secondary spectra with parent ion charge numbers in the range 0–5. The dynamic exclusion time for the tandem mass spectrometry scans was set to 30 s of seconds to avoid duplication of parent ion scans. The non-standard quantification method uses the LFQ quantification principle, where the relative quantification value of each sample is obtained from the LFQ intensity, which is corrected by the search library software (Cox et al., 2014).

### KOG/COGs and gene ontology annotation

To predict and categorize the potential uses of the unigene products, the KOG/COGs (clusters of orthologous groups) of the proteins were aligned to the entries in the EggNOG database (http://www.ncbi.nlm.nih.gov/COG/) (Galperin et al., 2015).

Gene ontology (GO) annotation is to annotate and analyze the identified proteins using EggNog-Mapper software (V2.0). The software extracts the GO ID from each protein annotation result based on the EggNOG database and then categorizes the protein function based on cell component, molecular function, and biological process.

### KEGG pathway annotation

KEGG connects data on known molecular interaction networks, including pathways and complexes (“Pathway” databases), information on genes and proteins derived from genomic projects (including gene databases), and information on biochemical compounds and reactions (including compound and reaction databases) (Kanehisa et al., 2016). Metabolism, genetic information processing, environmental information processing, cellular processes, murine diseases, and drug development are the main topics covered by KEGG pathways. Protein pathways were annotated using the KEGG database.

### Subcellular localization

An improved version of PSORT/PSORT II called WoLF PSORT was applied to the problem of predicting the sequence of eukaryotic organisms (Horton et al., 2007). WoLF PSORT converts the amino acid sequence of a protein into a digital localization feature; based on sequencing signals, amino acid composition and functional motifs (e.g., DNA binding motifs). After conversion, predictions are made using a simple K-nearest neighbor classifier. To research prokaryotic species, the subcellular localization prediction software known as CELLO was utilized (Yu et al., 2004).

### RNA extraction and qPCR

Following the instructions of a commercial kit (LS1040, manufactured by Promega in the United States), total RNA was extracted and purified before being utilized as a template in a cDNA reverse transcription synthesis manufactured by a commercial kit (K1622, manufactured by Thermo in the United States). The conditions were as follows: 42°C for 30 min and 85°C for 10 min. Real-time fluorescence quantitative PCR (qPCR) was performed using an UltraSYBR Mixture (Low ROX, CW2601, Cwbio, China), and a Stratagene Mx3000P (Agilent, United States); the reaction conditions were 95°C for 3 min, 95°C for 15 s, 55°C for 30 s, and 72°C for 30 s, with 40 cycles. The primer gene sequences are shown in Table 2.

**TABLE 2:** Sequences of primers used in qPCR.


Serpina3n F: 5CCT​CGG​GAC​ACA​TTC​CAG​TC3
Serpina3n R: 5CCA​CAA​CAG​TGC​AGT​TCA​GC3
Lst1 F: 5AAG​ACA​TGG​GGC​TGT​AAG​GAT3
Lst1 R: 5TCA​CTC​TCT​GTA​AAC​CAG​AT3
Srgn F: 5GGG​TCC​GCT​GAT​AGA​ACC​GCC3
Srgn R: 5CAA​AAC​AGG​ATC​GGT​CAT​CGG3
Fgb F: 5CCA​GCC​AAA​GTT​GAT​GCA​GG3
Fgb R: 5ACG​CAA​CTC​ACA​CCC​TGC​AGG3
Saa4 F: 5GTC​ACC​GTC​ATT​ATC​CTC​TGC​T3
Saa4 R: 5TCT​GCA​CAA​GTC​CCA​AGT​CC3
Ldhb F: 5GAA​CCC​AGA​GAT​GGG​AAC​GG3
Ldhb R: 5TTC​GAT​GAG​GTC​AGC​CAC​AC3

### Western blotting analysis

For separation using sodium dodecyl sulfate-polyacrylamide gel electrophoresis (SDS-PAGE), 20 g of protein/well was loaded onto a 10% gel. Polyvinylidene difluoride (PVDF) membranes (0.45 or 0.20 m pore size; Millipore, Billerica, MA, United States) were used to transfer gel electrophoresis. Lotted membranes were sealed with 5% skim milk powder in a Tris-buffered salt solution (25 mM Tris, pH 7.5 and 150 mM NaCl) containing 0.05% Tween 20 (TBST) for 2 h at room temperature. Then, for 4 h at room temperature, the membranes were incubated with a diluted primary antibody against the target protein. The membrane is incubated for 2 h at room temperature with the appropriate dilution of secondary antibody coupled to horseradish peroxidase after being washed in TBST solution for 10 min. Thermo Scientific (Waltham, MA, United States) ECL chemiluminescent reagents were used to see protein blots. GAPDH concentrations served as the top-sample control.

### Statistical analysis

The mean ± standard deviation is used to express all data in this study. Plotting and statistical analysis was done using GraphPad Prism, version 9.0 (GraphPad Software, La Jolla, CA, United States). The t-test was employed to compare groups. The following values were regarded as statistically significant: *p < 0.05, **p < 0.01, ***p < 0.001.

## Result

### JG improved the expression of bone metabolic biomarkers

**FIGURE 1:**
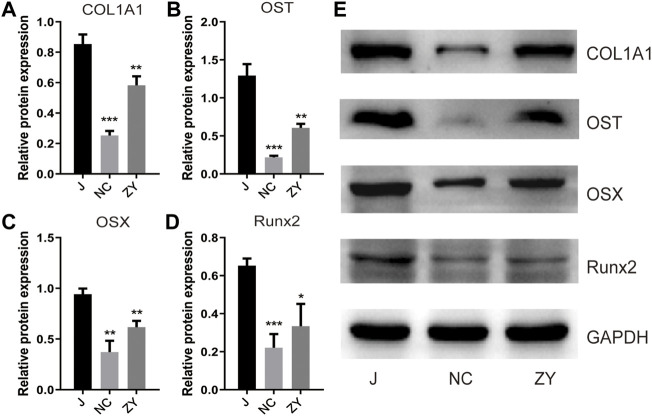
JG improved the expression of bone metabolic biomarkers. Expression of type I collagen (Col1a1). **(A)**, osteocalcin (OST). **(B)**, Osterix (OSX). **(C)**, and Runx2. **(D)** in each group by western blot analysis. **(E)**. The data are presented as the mean ± standard deviation, with n = 3 samples for each group. *p < 0.05, **p < 0.01, ***p < 0.001.

To determine the success of OVX rat model construction and to investigate the changes of bone metabolic indexes after JG treatment, detection of the Col1a1, osteocalcin, Osterix, and Runx2 protein expression levels by western blot (Figure 1E).

According to the findings of Western blot analysis, the protein expression of Col1a1 (Figure 1A), osteocalcin (Figure 1B), Osterix (Figure 1C), and Runx2 (Figure 1D) decreased in the NC group and increased after drug treatment compared to the J group. These results confirm that the JG is effective in treating the osteoporosis that occurs in OVX rats.

### 4D label-free quantitative proteomics analysis among the J, NC, and ZY group with proteomic data

**FIGURE 2:**
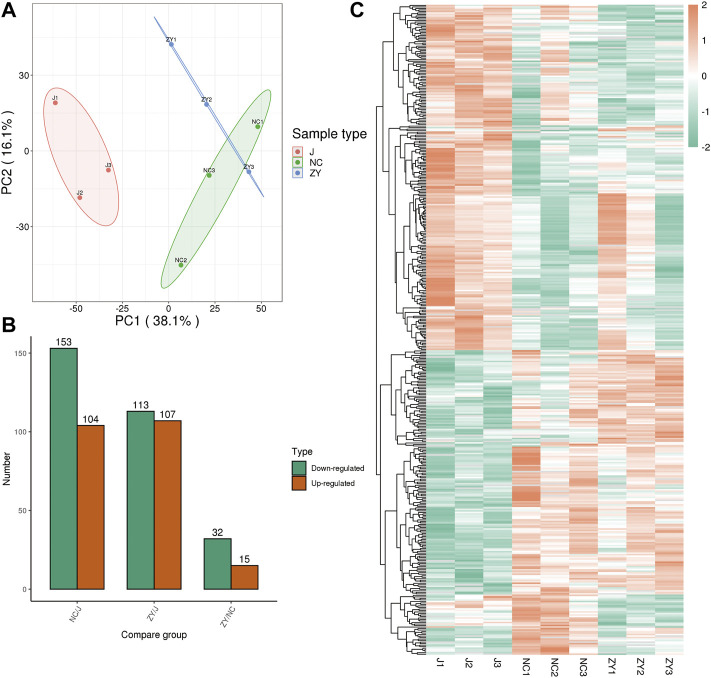
4D label-free quantitative proteomics analysis of differentially expressed proteins (DEPs) among the different groups. **(A)** PCA score plots of proteome data. **(B)** Number of DEPs between each group. **(C)** Heat map of DEPs between each group.

The tissue samples from rat vertebrae (L3, L4) that were used in our research were analyzed using high-throughput, label-free liquid chromatography-tandem mass spectrometry (LC-MS/MS) experiments. These experiments identified a total of 5054 quantifiable proteins in these samples. The results of the unsupervised principal component analysis (PCA) showed the separation between the experimental group (NC group and ZY group) and the J group (Figure 2A).

The proteins were selected with an FC > 1.5, and a statistically significant difference in the DEPs between the two groups was indicated by a value of *p* < 0.05. There were 104 up-regulated and 153 down-regulated differentially expressed proteins (DEPs) in the NC group vs. J group (Figure 2B) and 107 up-regulated and 113 down-regulated DEPs in the J group vs. ZY group (Figure 2B). In contrast, there were 15 up-regulated and 32 down-regulated DEPs in the ZY group vs. NC group (Figure 2B). Heatmap depicted the differential protein expression between each group (Figure 2C).

### Identification of differential proteins associated with PMOP

**FIGURE 3:**
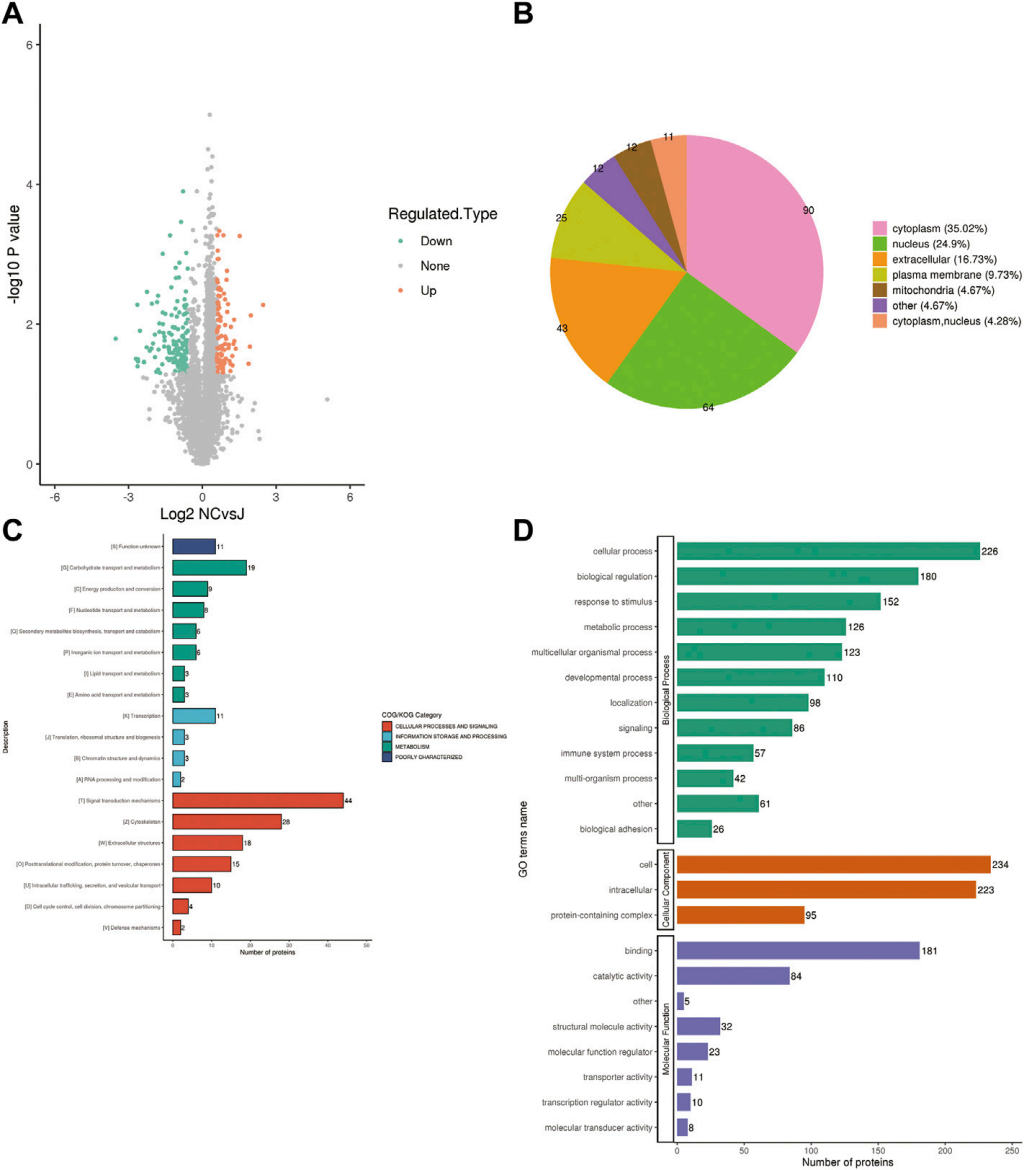
**(A)** A volcano plot depicting the DEPs between the NC group and the J group when FC > 1.5. **(B)** Subcellular localization of DEPs between NC group vs. J group. **(C)** COG/KOG analysis of DEPs between NC group vs. J group. **(D)** GO annotations of DEPs between NC group vs. J group.

To further determine which proteins are responsible for the PMOP, we analyzed the DEPs between the NC group vs. the J group. The volcano plot showed differential proteins, and there were 104 upregulated and 153 downregulated DEPs between NC vs. J (Figure 3A). WoLF PSORT was used to correctly predict the subcellular localization of DEPs, and it found that the majority of the proteins are found in the cytoplasm (35.02%), the nucleus (24.9%), and the extracellular (16.73%) (Figure 3B).

Based on the EggNOG Database, Clusters of Orthologous Groups of protein/Eukaryotic Orthologous Groups (COG/KOG) annotations were conducted. To make a prediction regarding the functional classification of DEPs, a COG/KOG analysis was performed (Figure 3C). Even though the function of 11 proteins was still unknown, the catalog of 18 known functions revealed that signaling transduction mechanisms (44 proteins) were the most abundant category, followed by cytoskeleton (28 proteins), carbohydrate transport and metabolism (19 proteins) and so on.

**FIGURE 4:**
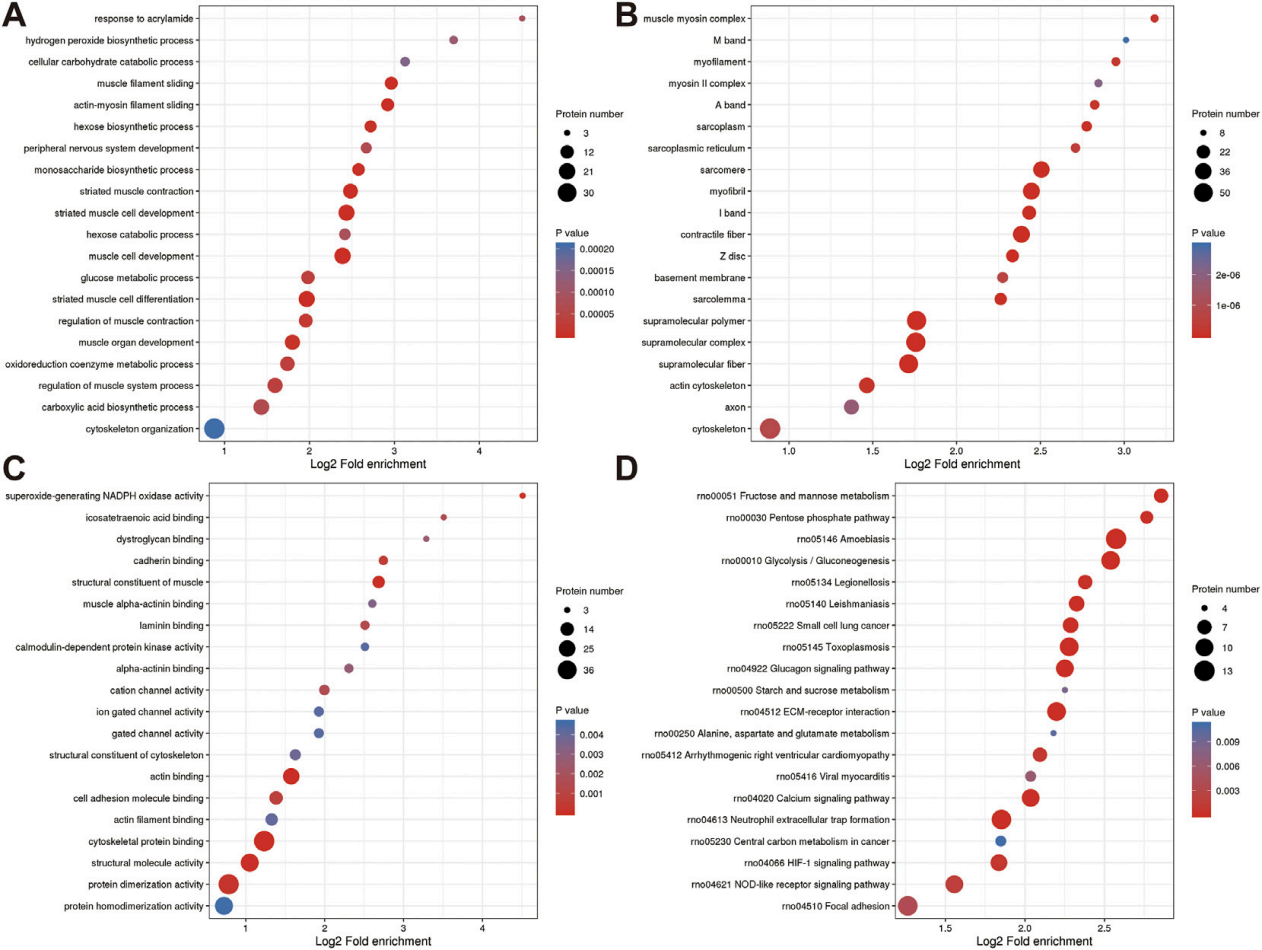
Annotation term levels of DEPs between NC group vs. J group are indicated as biological process **(A)**, cellular component **(B)**, molecular function **(C)**, and KEGG pathway **(D)**.

The proteins that were identified in terms of the components of the cell were classified using GO analysis. According to the results of the GO functional classification, DEPs were classified as belonging to a total of 23 GO terms, the majority of which were associated with 12 biological processes, 2 cellular components, and 8 molecular functions (Figure 3D). The “cellular process” component of the biological process contained 226 proteins, followed by the “biological regulation” component, which contained 180 proteins, and then the “response to stimulus” component, which contained 152 proteins, etc. “Cell” and “intracellular” were the two terms that appeared the most frequently in the cellular component, each accounting for 234 and 223 proteins, respectively, followed by “protein-containing complex” (95 proteins). In addition, “binding” (181 proteins) and “catalytic activity” (84 proteins), are the top two ranked terms in molecular function. The roles of DEPs in PMOP and the impact of estrogen deficiency on PMOP in rats were investigated using functional enrichment analyses, which included GO annotation and the KEGG pathway (Figure 4). Some DEPs were enriched for “striated muscle contraction”, “striated muscle cell development”, “striated muscle cell differentiation”, “regulation of muscle contraction”, and “muscle organ development”.

Additionally, DEPs were enriched for terms like “sarcomere,” “myofibril,” and “contractile fiber” that are associated with muscles. KEGG pathway analysis revealed that several pathways including “Glucagon signaling pathway (Xie et al., 2021)”, “ECM-receptor interaction (Gautvik et al., 2020)”, “Calcium signaling pathway (Yang et al., 2021)”, “Neutrophil extracellular trap formation (Flores et al., 2016)”, “HIF-1 signaling pathway”, and“NOD-like receptor signaling pathway (Tao et al., 2021)” (Figures 4A–D).

### Identification of differential proteins associated with JG for PMOP

**FIGURE 5:**
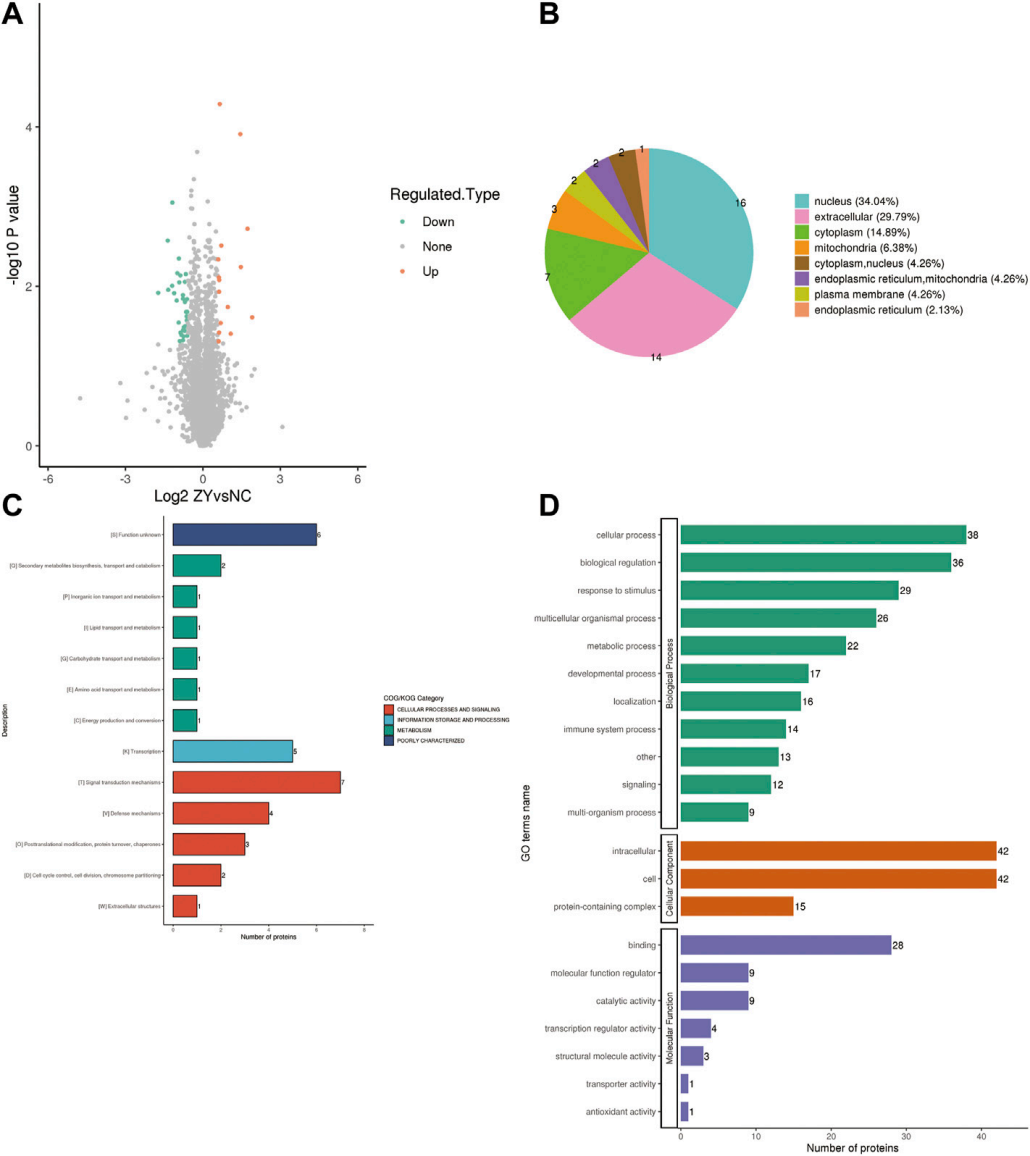
**(A)** A volcano plot depicting the DEPs between the ZY group and NC group when FC > 1.5. **(B)** Subcellular localization of DEPs between ZY group vs. NC group. **(C)** COG/KOG analysis of DEPs between ZY group vs. NC group. **(D)** GO annotations of DEPs between ZY group vs. NC group.

To further explore which proteins are the targets of JG for PMOP treatment, we analyzed the DEPs between the ZY group and the NC group. The volcano plot showed differential proteins, with 15 upregulated and 32 downregulated DEPs between ZY vs. NC (Figure 5A).

WoLF PSORT predicted the subcellular localization of DEPs, and most of the proteins are located in the nucleus (34.04%), extracellular (29.79%), and cytoplasm (14.89%) (Figure 5B). COG/KOG analysis was carried out to forecast the functional classification of DEPs using the EggNOG Database (Figures 5C,D).

**FIGURE 6:**
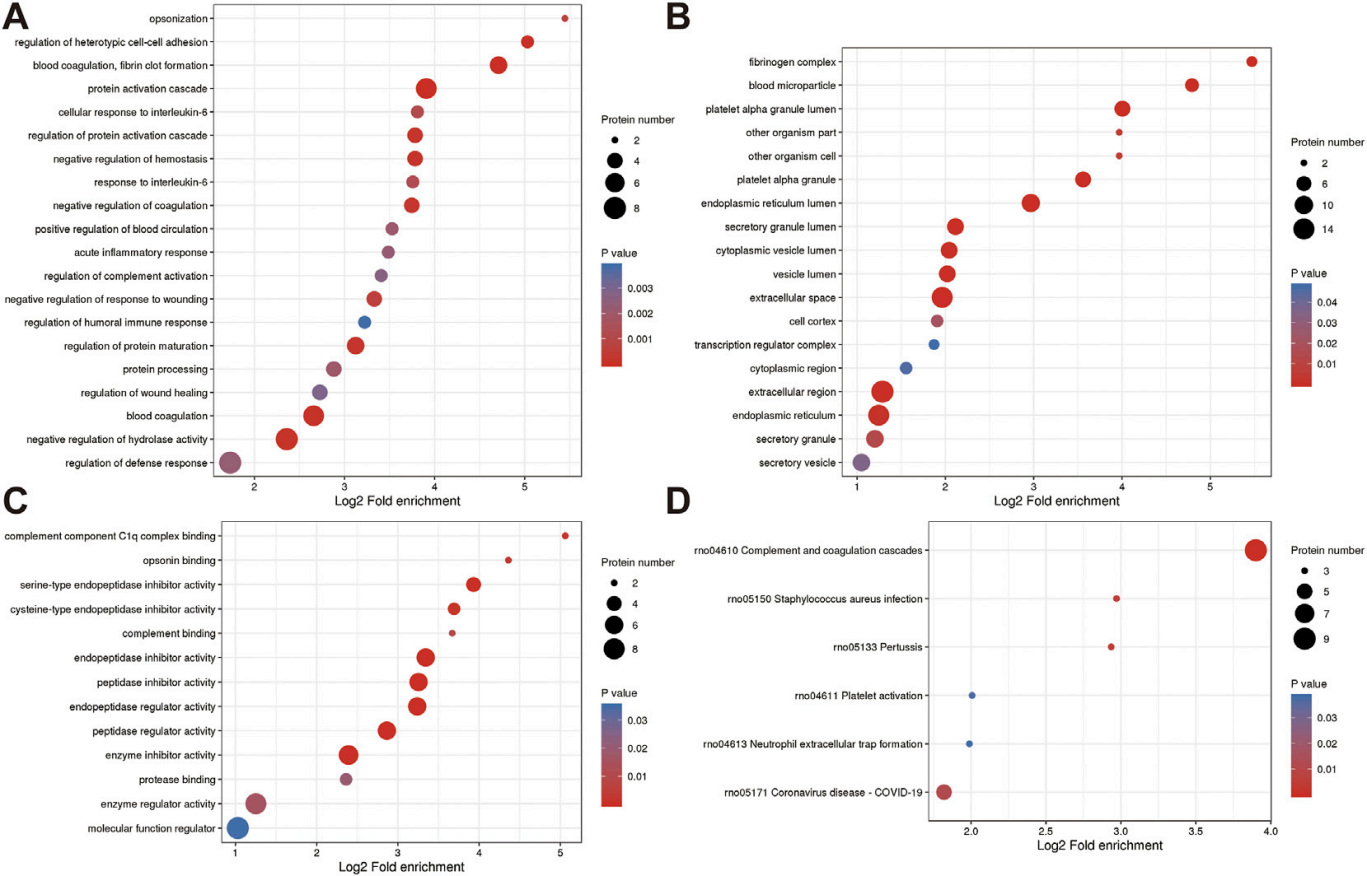
Annotation term levels of DEPs between ZY group vs. NC group are indicated as biological process **(A)**, cellular component **(B)**, molecular function **(C)**, and KEGG pathway **(D)**.

Even though the function of six proteins was unknown, the catalog of 12 known functions revealed that the largest category was signaling transduction mechanisms (7 proteins). This was followed by transcription (5 proteins), defense mechanisms (4 proteins), Posttranslational modification, protein turnover, chaperones (3 proteins), and so on. Analysis of the response of DEPs to JG treatment using gene ontology (GO) term enrichment. GO functional classification revealed that DEPs were classified in 21 GO terms, including 11 biological processes, 3 cellular components, and 7 molecular functions (Figures 6A–D).

In the biological process, 38 proteins were involved in the “cellular process”, which is followed by 36 proteins in “biological regulation” and 29 proteins in “response to stimulus”. In the cellular components, the two most abundant terms were “intracellular” and “cell”, both with 42 proteins, followed by “protein-containing complex” (15 proteins). Moreover, “binding” (28 proteins), “molecular function regulator” (9 proteins), and “catalytic activity” (9 proteins), were the top three terms in molecular function.

The roles of DEPs in JG for PMOP treatment and the impact of JG on PMOP in rats were examined using functional enrichment analyses, which included GO annotation and the KEGG pathway (Figure 5). The significant terms of GO functional enrichment and KEGG pathways were shown in Figure 6. Some DEPs were enriched for “protein activation cascade”, “cellular response to interleukin-6”, “regulation of protein activation cascade”, and “response to interleukin-6”.

Furthermore, DEPs were also enriched in terms related to mitochondria such as “fibrinogen complex”, “blood microparticle”, “endoplasmic reticulum lumen”, “secretory granule lumen”, “cytoplasmic vesicle lumen”, “vesicle lumen”, “extracellular space”, “extracellular region”, “endoplasmic reticulum”, “Endopeptidase inhibitor activity”, “peptidase inhibitor activity”, “endopeptidase regulator activity”, “peptidase regulator activity” and “enzyme inhibitor activity”. KEGG pathway analysis revealed the involvement of differential proteins in the “complement and coagulation cascade” after JG treatment (Figure 6D).

### Validation of target proteins associated with JG for PMOP treatment

**FIGURE 7:**
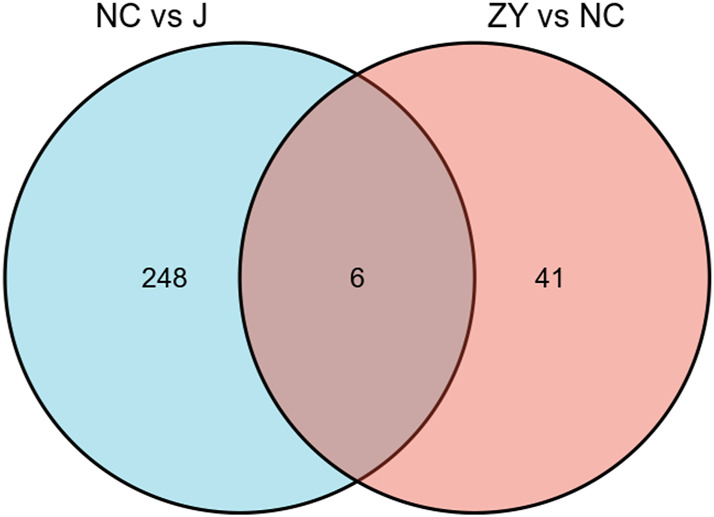
The Venn diagram indicated a comparison of DEPs between the NC group vs. J group and ZY group vs. NC group.

As demonstrated in a Venn diagram (Figure 7), 6 of the proteins were common for both groups. 248 proteins were found only in NC vs. J, and 41 proteins in ZY vs. NC. In this study, l-lactate dehydrogenase B chain (Ldhb), Serine protease inhibitor A3N (Serpina3n), Leukocyte-specific transcript 1 (Lst1), Serglycin (Srgn), Fibrinogen beta chain (Fgb) and Hermansky-Pudlak syndrome 5 protein homolog (Saa4) all expressed in two groups (Table 3).

**TABLE 3:** Changes in the tissue of rat vertebrae proteins between NC group vs. J group and ZY group vs. NC group.

Protein accession	Gene name	Protein description	Average J	Average NC	Average ZY	NC/J ratio	NC/J *p* value	ZY/NC ratio	ZY/NC *p* value
P42123	Ldhb	L-lactate dehydrogenase B chain	1.374797	0.647097	0.978106	0.471	0.001558475	1.512	0.004604846
A0A0H2UHI5	Serpina3n	Serine protease inhibitor A3N	0.693351	1.773372	0.533277	2.558	0.022459868	0.301	0.012064865
A0A0U1RRR5	Lst1	Leukocyte-specific transcript 1	0.657299	1.469296	0.873406	2.235	0.010949046	0.594	0.042487841
P04917	Srgn	Serglycin	0.79455	1.587644	0.617805	1.998	0.001729083	0.389	0.002674471
P14480	Fgb	Fibrinogen beta chain	0.793743	1.391916	0.814341	1.754	0.04188621	0.585	0.047580928
Q7TMC3	Saa4	Hermansky-Pudlak syndrome 5 protein homolog	0.653504	1.604794	0.741702	2.456	0.017412435	0.462	0.012185175

For validation of the 6 protein expression levels, we further examined the mRNA expression levels corresponding to the above 6 genes with qPCR. The results of the qPCR performed on six different DEPs were in agreement with the findings of the proteomic analysis.

**FIGURE 8:**
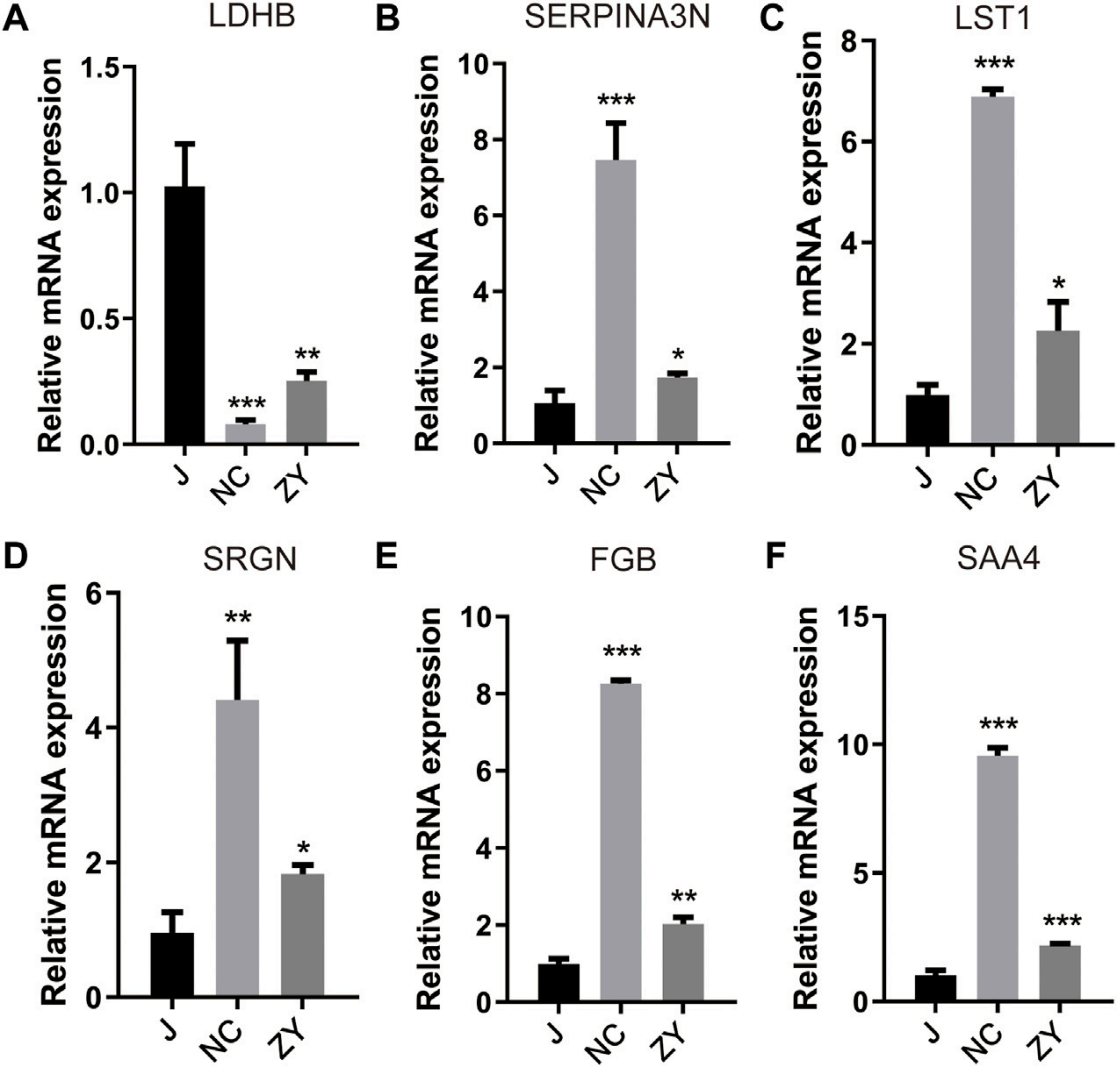
Differential expression levels for selected genes [LDHB **(A)**, SERPINA3N **(B)**, LST1 **(C)**, SRGN**(D)**, FGB **(E)** and SAA4 **(F)**] were validated by qPCR.

As expected, ovariectomy decreased Ldhb which increased after JG treatment (Figure 8A). Serpina3n, Lst1, Srgn, Fgb, and Saa4 expression was further increased after ovariectomy, and the expression of the above five proteins decreased after JG treatment (Figures 8B–F). Our data suggest that JG may achieve therapeutic effects by modulating the expression of the above key proteins.

### GO and KEGG enrichment analysis was performed on six target protein-coding genes with differential expression.

To gain a deeper understanding of the functions of six target proteins following JG treatment, GO and KEGG analyses were carried out to determine the roles that each of these target proteins play.

Enrichment analysis of the GO-represented biological processes and molecular functions revealed that these key genes were potentially involved in a wide range of metabolic pathways and regulatory processes. The results of an enrichment analysis of the biological processes and molecular functions that were represented by GO showed that these target genes had the potential to be involved in a wide variety of metabolic pathways and regulatory processes concerning the PMOP. Ldhb may engage in cofactor binding and oxidation-reduction processes (Wu et al., 2017; Zhong et al., 2017; Shen et al., 2022). The results showed that Serpina3n participated in the inflammatory response, and cellular response to interleukin-6 and interleukin-1, which suggested that this protein may play a role in the regulation of inflammatory factors. Lst1 functioned in immune response and developmental process. Srgn has a negative regulatory effect on ossification, and the pathogenesis of PMOP is likely related to this pathway. Fgb plays a major role in the secretion and transport of peptides and proteins. Saa4 engaged in response to stimulus and multicellular organismal process. The results of the above enrichment analysis suggested to us that JG may affect bone metabolism through the above-mentioned proteins for therapeutic purposes.

## Discussion

PMOP is a metabolic bone disease with reduced bone formation, increased bone resorption, degradation of bone tissue microstructure, and reduced bone strength due to postmenopausal ovarian hypofunction (Maeda and Lazaretti-Castro, 2014; Kataoka et al., 2020). The physical and mental health of many women, as well as the quality of their lives, have been seriously threatened by the high incidence of PMOP and its accompanying pain and fractures.

The decrease in estrogen levels after menopause causes a decline in osteogenic differentiation, bone resorption is greater than bone formation, and an imbalance in bone homeostasis is one of the crucial factors of postmenopausal osteoporosis (Kim et al., 2020; Kou et al., 2020). Osteoblast differentiation as a key link in bone formation is important for the prevention and treatment of PMOP; therefore, promoting osteoblast differentiation can serve as a starting point for understanding how Chinese medicine can be used to prevent and treat PMOP (Zhou et al., 2022).

The regulation of the equilibrium between bone formation and bone resorption allows for the continuous remodeling and maintenance of bone mass, and this balance of bone metabolism is mainly mediated by osteoblasts and osteoclasts, and bone loss occurs when bone metabolism is out of balance. In postmenopausal women, estrogen deficiency leads to abnormal activation of osteoclasts and inhibition of osteoblast differentiation, which is the main reason why postmenopausal women are prone to osteoporosis (Teitelbaum, 2000; Marie, 2013).

According to traditional Chinese medicine, the primary pathogenesis of PMOP is kidney deficiency, which also involves spleen deficiency (Wang et al., 2010; Li et al., 2011). The Chinese botanical formula JG has demonstrated excellent effectiveness in the clinical treatment of PMOP by enhancing kidney and spleen function (Huang et al., 2017). JG can effectively improve the bone density of female rats, bone physical properties, and other bone metabolism indexes, which have promoted bone formation. However, the underlying mechanism of JG’s roles in PMOP still needs to be further explored.

Proteomics analysis was used in this study to thoroughly screen proteins that were differentially expressed, and bioinformatics analysis was used to assess the pathways and biological processes involved in osteoblast differentiation in the PMOP and JG treatments. Therefore, to illustrate the mechanism of JG treatment for PMOP, we designed experiments from the perspective of JG in regulating osteoblast differentiation. First, we established a postmenopausal osteoporosis model in rats by removing ovaries, and measured bone metabolism indexes such as Col1a1, and OST to confirm the therapeutic effect of JG in the treatment of PMOP. In terms of regulating osteoblast differentiation, we again demonstrated that JG has a regulatory effect on osteogenic transcription factors such as OSX and Runx2 in the process of osteogenic differentiation.

After confirming the efficacy of JG in model rats, we further investigated the changes in protein expression levels in rats between treatments to explore the potential mechanism of JG regulation of osteoblasts.

The recently developed 4D label-free quantitative proteomics is a high-throughput proteomics technology that enables faster, more sensitive, and more accurate identification and quantification of various proteins (Meier et al., 2018). This study aimed to obtain proteomic profiles of JG-treated OVX rats using a 4D-label-free based approach and bioinformatics analysis of three groups of rat vertebral tissues, as well as to explore the potential molecular mechanisms of JG in the treatment of PMOP. Our study employed a 4D label-free quantitative method for proteomic analysis of the J group, NC group, and ZY group.

We identified 5054 quantifiable proteins in the tissue samples from rat vertebrae in our research. The PCA analysis is used to test the statistical consistency of quantitative results from biological replicate samples. By taking the ratio of the mean relative quantitative values of each protein in multiple replicate samples as the Fold Change (FC) of the difference, we determined that FC > 1.5 was a significant difference and DEP screening was performed by selecting proteins with FC > 1.5 between the two groups. The results revealed that there were 104 upregulated and 153 downregulated DEPs in the NC group vs. J group and there were 15 upregulated and 32 downregulated DEPs in the ZY group vs. NC group.

We screened the differential proteins in the sham-operated group versus the PMOP model group (group NC) against the differential proteins in the treated group (group ZY) versus the PMOP model group (group NC) after treatment with JG and found that six of the differential proteins were altered in expression in all three groups. This also suggested to us that these 6 key proteins may be the target proteins for JG treatment of PMOP. This offers a foundation for identifying the precise mechanism of JG treatment for PMOP and investigating the pathogenesis linked to abnormalities in osteoblast differentiation in the field of PMOP research.

In this study, Ldhb, Serpina3n, Lst1, Srgn, Fgb, and Saa4 were expressed in both groups. To confirm the expression levels of these six proteins, we further examined the mRNA expression levels corresponding to the above six genes by qPCR. qPCR results for the six DEPs were consistent with 4D label-free quantitative proteomics analysis and, as expected, ovariectomy reduced Ldhb, whereas Ldhb increased after JG treatment. The expression of Serpina3n, Lst1, Srgn, Fgb, and Saa4 was further increased after ovariectomy, whereas the expression of the above five proteins was decreased after JG treatmen.

Both the pathogenesis of PMOP and the JG treatment of PMOP may be significantly affected by these proteins. We used bioinformatics tools to analyze these proteins and explore the role played by these six target proteins in the pathogenesis of PMOP. According to the results of GO and KEGG analysis, it was found that three of these six proteins (Ldhb, Serpina3n, and Lst1) have a regulatory role in regulating osteoblast differentiation, which may provide ideas to explain the role of JG in promoting osteoblast differentiation in PMOP patients.

The results of both 4D label-free quantitative proteomics analysis and qRT-PCR illustrated that Ldhb expression was decreased in OVX rats and upregulated after JG treatment. In osteoblast aerobic glycolysis, the interconversion between lactate and pyruvate is catalyzed by lactate dehydrogenase (LDH); where the isomer Ldhb has a higher affinity for lactate (LA) (Doherty and Cleveland, 2013; Valvona et al., 2016) and prefers to convert lactate to pyruvate compared to LDHA (De La Cruz-Lopez et al., 2019). It has been previously demonstrated that LA, as a metabolic intermediate, can induce osteoblast differentiation upon entry into cells, that LA can be converted to pyruvate by Ldhb, and that osteoblast differentiation can be induced by oxidative metabolism in part by stabilizing HIF1α expression (Wu et al., 2017; Shen et al., 2022), and that this osteoblast differentiation-inducing effect of lactate can be inhibited by interfering with its metabolism using Ldhb -specific siRNA. The above results suggest that Ldhb plays a key role in osteoblast differentiation and that decreased expression of Ldhb may lead to destabilization of HIF1α expression and thus inhibition of osteoblast differentiation, whereas Ldhb expression levels increased after treatment with JG, which also suggests that Ldhb may be a target protein in the mechanism of JG-induced osteoblast differentiation.

Serpina3n is a homolog of human SERPINA3 (human α-1 antitrypsin, α1-ACT) in rodents and belongs to the evolutionary branch of the serine protease inhibitor (serpin) superfamily A. e Previous studies on Serpina3n have shown that Serpina3n is significantly expressed in female mouse osteoblasts and inhibits the differentiated mouse phenotype of osteoblasts (Ishida et al., 2018). The mRNA levels of Runx2, ALP, osteocalcin, and Col1a1 are markedly increased in female osteoblasts when endogenous Serpina3n levels are reduced by small interfering RNA, enhancing osteoblast differentiation.

Interestingly, in OVX rats, the lack of estrogen leads to the upregulation of inflammatory factors such as IL-6, TNF-α, and IL-1β expression, which are important factors in inhibiting osteoblast differentiation, but the exact mechanism of inflammatory factors such as inhibition of osteoblast differentiation is still unexplored. GO and KEGG analysis showed that Serpina3n is involved in the response to IL-6, TNF-α, IL-1β responses, and upregulation of inflammatory factors can induce Serpina3n expression *in vitro* and *in vivo*, and the above results provide ideas to further explore the exact mechanisms by which inflammatory factors inhibit osteoblast differentiation.

Compared with the sham-operated group, Serpina3n expression was elevated in OVX rats and decreased after JG treatment. This evidence suggests that the Serpina3n protein plays an important role in the promotion of osteoblast differentiation by JG, and JG promotes osteoblast differentiation by down-regulating Serpina3n expression. This also suggests that Serpina3n is a key target for JG to exert its anti-osteoporosis effect.

IFN-γ, TNF-α, IL-1, and LPS significantly increased the expression of the Lst1 transcript in human intestinal cells in response to inflammatory stimuli. By using FACS and Western blot analysis, it was also discovered that the protein levels of Lst1 isoforms were increased in response to inflammatory stimuli (Heidemann et al., 2014). Furthermore, previous studies have observed results consistent with some of these findings, finding that the full-length Lst1 heterodimer is induced by inflammatory stimuli (IFN-γ and TNF) in HeLa cells (Schiller et al., 2013). Chronic inflammation, which is brought on by an estrogen deficit, results in increased levels of inflammatory factors, resulting in increased expression of Lst1 in OVX rats and decreased expression after treatment with JG, which also suggests that Lst1 may serve as a potential drug target for JG treatment. It has been suggested that Lst1 deficiency in osteoblasts may also lead to functional alterations and defective trabecular bone formation, but the exact mechanism has not yet been investigated, especially whether Lst1 may affect osteoblast differentiation needs to be further investigated.

The roles played by Srgn, Fgb, and Saa4 in osteoblasts compared to Ldhb, Serpina3n and Lst1 have not been reported in the literature, and although changes in their expression have been analyzed in OVX rats and JG-treated rats, their exact mechanisms in PMOP remain unknown and need to be further explored. In addition, we still have some limitations in our experimental design. Although the doses of JG we are currently using are experimentally proven to be appropriate, we will need more concentration gradients in the future to rule out the effects of single concentration gradients and high doses of the drug on experimental results (Huang et al., 2017; Sun et al., 2022). In our subsequent experiments, we will investigate the effect of the composition of the main components of JG on the overall efficacy of JG in different proportions to refine our results.

In summary, based on earlier findings, the current study discovered that treatment with JG in OVX rats increased the expression of genes related to osteoblast transcription factors, including Runx2 and OST, which encourage osteoblast differentiation. The combined “multi-component, multi-target” action of traditional Chinese medicine is consistent with the impact of JG on multiple protein targets during osteoblast differentiation. We speculate that DEPs are potential drug targets related to PMOP; however, the limitation is that no study has shown that JG can exert anti-osteoporosis effects by regulating six proteins related to osteoblast differentiation, and the mechanism of action of these six potential drug targets in osteoporosis needs to be further investigated. Using osteoblasts as the target, it is important to reveal the mechanism of action of JG in the treatment of PMOP for the future selection of JG in the clinical treatment of PMOP.

## Conclusion

The mechanism of JG’s role in the treatment of PMOP was investigated using 4D label-free quantitative proteomics analysis and proteomic microarray technology. The results showed that JG is involved in regulating target proteins associated with osteoblast differentiation through. This supports our research hypothesis that JG regulates osteoblast differentiation as well as the findings of some earlier studies. Specifically, JG controls a variety of target proteins that are involved in signal transduction and have therapeutic effects *via* a variety of signaling pathways. We also found a relationship between differentially expressed proteins such as Ldhb, Serpina3n, Lst1, Srgn, Fgb, Saa4, and PMOP. Some studies have shown that Ldhb, Serpina3n, and Lst1 play a regulatory role in osteoblast differentiation, however, the remaining three target proteins, Srgn, Fgb, and Saa4, affect PMOP onset and development is unclear; the specific mechanism of how these six differential proteins are involved in the transduction of signaling pathways to regulate osteoblast differentiation deserves further investigation.

## Data Availability

The datasets presented in this study can be found in online repositories. The names of the repository/repositories and accession number(s) can be found below: ProteomeXchange (http://proteomecentral.proteomexchange.org/cgi/GetDataset), PXD037316.
