# Supplementary material for: 4D label-free quantitative proteomics analysis to screen potential drug targets of Jiangu Granules treatment for postmenopausal osteoporotic rats
Source: Front Pharmacol. 2022 Nov 1;13:1052922. doi: 10.3389/fphar.2022.1052922 (PMC9663813; doi:10.3389/fphar.2022.1052922)
Supplement: Supplementary file 1 [file DataSheet2.docx]

**Dosage calculation**

1. Yin yanghuo 12g, Gu suibu 12g, Lu xiancao 12g, Dang shen 10g, Jiang huang 10g, Shan zhuyu 9g, Gou qi 9g, Huai shan 9g, Chen pi 6g, Xi honghua 2g

Total 91g/ dose*day

Calculation process:

1. Calculate the dosage required by the person;

2. Calculate the body surface area of human and rat according to the conversion formula of body surface area of human and rat;

3. Convert unit of raw medicine quantity;

4. Calculate the daily dosage of each rat;

5. Calculate the total amount of medication.

Calculation of dose of rats (calculated by body surface area)

Body surface area：A=K*(w^2/3^/10000)

A: Body surface area (m^2^) K：Body surface area coefficient w：weight (g)

Known：a. Standard weight: w_human_=70kg =70000g; w_rat_=0.2kg=200g

K_human_=10.500 K_rat_=9.100

b. the amount raw medicine per person/day =91g，

1. Calculation of the amount of raw medicine required per kg of human standard body weight

Dose (human g/kg) =91g/w_human_= 91g/70kg =1.300g/kg

2. A_human_=10.5* (70000^2/3^/10000) =1.783m^2^

A_rat_=9.1* (200^2/3^/10000) =0.031m^2^

3. Conversion of the unit of raw drug required per kg of standard body weight from g/kg to g/m2

Dose (human g/m^2^) = dose (human g/kg) * w_human_ / A_human_

= 91g/70kg * 70kg/1.783m^2^

= 51.038g/m^2^

5. Calculation of the amount of raw medicine required per kg of rat standard body weight

Dose(rat)=51.038g/m^2^*A_rat_/w_rat_

=51.038g/m^2^ *0.031m^2^/0.2kg

=7.911g/kg

known that the average body weight of female SD rats aged from 3 to 6 months was 300g.

A total of 144 copies of drugs were purchased: a total of 13104g was concentrated into 5L drug concentrate (concentration of 2kg/L).

13104g/7.911g/kg=5000ml/X (X=Concentrated liquid volume ml/kg）

X=3.019ml/kg=6.038g/kg

Dilution of concentrated solution 120g concentration /80ml water

60ml/(60+80)ml=3.019ml/kg/N (N= Lavage coefficient）

N=7.044ml/kg
