# Supplementary material for: 4D label-free quantitative proteomics analysis to screen potential drug targets of Jiangu Granules treatment for postmenopausal osteoporotic rats
Source: Front Pharmacol. 2022 Nov 1;13:1052922. doi: 10.3389/fphar.2022.1052922 (PMC9663813; doi:10.3389/fphar.2022.1052922)
Supplement: Supplementary file 2 [file DataSheet1.docx]

Morphological identification

1. Yinyanghuo

*Epimedium brevicornu Maxim. [Berberidaceae; Epimedii Folium]*

The leaflets are oval, 3-8cm long and 2-6cm wide; the apex is slightly pointed, the base of the terminal leaflet is heart-shaped, the leaflets on both sides are smaller and eccentric, the outer side is larger, and it is ear-shaped，with yellow thorn-like serrations on the edge; yellow-green upper surface, gray-green lower surface, 7-9 main veins, base with sparse slender hairs, veinlets protruding on both sides, network veins obvious; small petioles 1-5cm long. Leaves nearly leathery. Gas micro, slightly bitter taste.

2. Gusuibu

*Davallia mariesii Moore ex Bak. [Davalliaceae; Drynariae Rhizoma]*

The product is flat and long, mostly curved, with branches, 5-15cm long, 1-1.5cm wide, and 0.2-0.5cm thick. The surface is densely covered with dark brown to dark brown small scales, soft as hair, brown or dark brown when burned by fire, with raised or concave round leaf scars on both sides and on the upper surface, a few with petiole residues and fibrous roots remain. Body light, brittle, easy to break, reddish-brown cross-section, yellow dot-shaped vascular bundles, arranged in rings. Gas micro, taste light, slightly astringent.

3. Shanzhuyu

*Cornus officinalis Sieb. et Zucc. [Cornice; Corni Fructus]*

This product is irregular flake or capsule, 1-1.5cm long and 0.5-1cm wide. The surface is purple-red to purple-black, shrunken and shiny. Some have round persistent calyx scars at the top and fruit stem scars at the base. Soft. Gas slightly, sour, astringent, slightly bitter.

4. Dangshen

*Codonopsis pilosula (Franch.) Nannf. [Campanulace; Codonopsis Radix]*

This product is long cylindrical, slightly curved, 10-35cm long and 0.4-2cm in diameter. The surface is gray-yellow, yellow-brown to gray-brown, the root head has many verrucous protruding stem scars and buds, and the top of each stem scar is in the shape of a concave dot; there are dense annular horizontal stripes under the root head. The lower part is gradually sparse, and some reach half of the whole length. The cultivated products have few or no annular horizontal stripes; the whole body has longitudinal wrinkles and scattered horizontally long lenticel-like protrusions, and there are often black-brown gelatinous substances at the broken roots. The quality is slightly soft or slightly hard and slightly tough, the section is slightly flat, with cracks or radial texture, the bark is light brown to yellowish brown, and the xylem is light yellow to yellow. Has a special aroma, slightly sweet taste.

5. Shanyao

*Dioscorea opposita Thunb. [Dioscoreaceae; Dioscoreae Rhizoma]*

This product is an irregular thick slice, with uneven shrinkage, white or yellow-white cut surface, firm and brittle, powdery. Slight gas, light taste, slightly sour.

6. Chenpi

*Citri [Rutaceae; Citri Reticulatae Pericarpium]*

This product is often peeled into several petals, the bases are connected, and some are irregular flakes, 1-4mm thick. The outer surface is orange-red or reddish-brown, with fine wrinkles and concave punctate oil chambers; the inner surface is pale yellow-white, rough, with yellow-white or yellow-brown tendon-like vascular bundles. Slightly hard and brittle. Fragrant, spicy, bitter.

7. Xihonghua

*Crocus sativus L. [Iridaceae；Croci Stigma]*

This product is linear, three-branched, about 3cm long. Dark red, the upper part is wider and slightly flat, the top edge is irregularly toothed, there is a short fissure inside, and a small yellow style is sometimes left at the lower end. Light body, soft, no oily luster, brittle and easy to break after drying. Gas-specific, slightly irritating, slightly bitter taste.

8. Jianghuang

*Curcuma longa L. [zingiberaceae; Curcumae Longae Rhizoma]*

This product is oblong or irregular flakes with different sizes, 3-6cm long, 1-3cm wide and 0.1-0.4cm thick. The outer skin is gray-yellow, rough and shrunken, and sometimes links and fibrous root marks are visible. The cut surface is yellowish-white to brownish-yellow, with a ring pattern and many small veins. Crispy and firm. The cross section is grayish white to brownish yellow, slightly silty. The aroma is specific, the taste is slightly bitter and acrid.

9. Gouqi

*Lycium chinense Mill. [Solanaceae; Lycii Fructus]*

This product is spindle-like or oval, 6-20mm long and 3-10mm in diameter. The surface is red or dark red, with small protruding style scars at the top and white fruit stem scars at the base. The peel is flexible and shrinking; the pulp is fleshy and soft. Seeds 20-50, reniform-like, flat and warped, 1.5-1.9mm long, 1-1.7mm wide, with pale yellow or brownish-yellow surface. Gas micro, sweet.

10. Luxiancao

*Pyrola calliantha H. Andr. [Pyrolaceae; Pyrolae Herba]*

This product has slender rhizomes. Stems cylindrical or with longitudinal ribs, 10-30cm long. The leaves are basal, oblong or suborbicular, 2-8cm long, dark green or purple-brown, rounded or slightly pointed at the apex, entire or with sparse serrations, the edge slightly revolute, the upper surface sometimes white along the veins Marked, sometimes whitish on lower surface. There are 4 to 10 flowers in the raceme; flowers are semi-drooping, sepals 5, tongue-shaped or ovate-oblong; petals 5, caducous, stamens 10, anther base with small angle, apical hole dehiscent; style exposed, ringed protruding stigma disk. Capsule oblate, 7-10mm in diameter, 5 longitudinal fissures, with spider silk-like hairs on the edges of the lobes. Gas micro, taste light, slightly bitter.
